# Supplementary material for: Extensive folding variability between homologous chromosomes in mammalian cells
Source: Mol Syst Biol. 2025 May 6;21(7):735–75. doi: 10.1038/s44320-025-00107-3 (PMC12222873; doi:10.1038/s44320-025-00107-3)
Supplement: Supplementary file 14 — Expanded View Figures [file 44320_2025_107_MOESM14_ESM.pdf]

Expanded View Figures

Figure EV1. Strategy, methodology and evaluation of GAM-Phasing pipeline for allele-specific contact maps.

(**A**) Schematic overview of GAM data collection, quality control steps, and merging of replicates R1 and R2. (**B**) GAM-phaser pipeline. (**C**) Percentage of reads that were phased to each allele. Conflicting reads are reads containing SNPs from both alleles. (**D**) Percentage of phased positive windows in the entire segregation table for all F123 3NPs passed quality controls GAM samples. (**E**) Phasing efficiency between GAM and Hi-C. GAM efficiency is measured as phased windows divided by the total number of called windows, while Hi-C efficiency is calculated dividing phased ligation events to unique ligation events; reported phasing efficiency was obtained from (Giorgetti et al, 2016). Below, schematic of a phaseable Hi-C ligation event. (**F**) Number of informative contact entries in the phased F123 GAM dataset in comparison with phased Hi-C data collected for human GM12878 B-lymphoblastoid cells (Rao et al, 2014b), at all intrachromosomal distances and for distances up to 10 Mb. (**G**) Number of nuclear sections that are positive for the presence of two or one *Hoxb1* or *Hoxb13* locus detected by cryo-FISH using 40 kb fosmid probes ($n = 341$ *Hoxb1* loci, $n = 362$ *Hoxb13* loci, $n = 2584$ nuclear sections imaged; data source from Barbieri et al, 2017). (**H**) Percentage of locus pairs detected at least once and Kendall's τ coefficient values for different resolutions and different distances. These metrics were used to decide on optimal resolutions of the maps.

 © The Author(s)

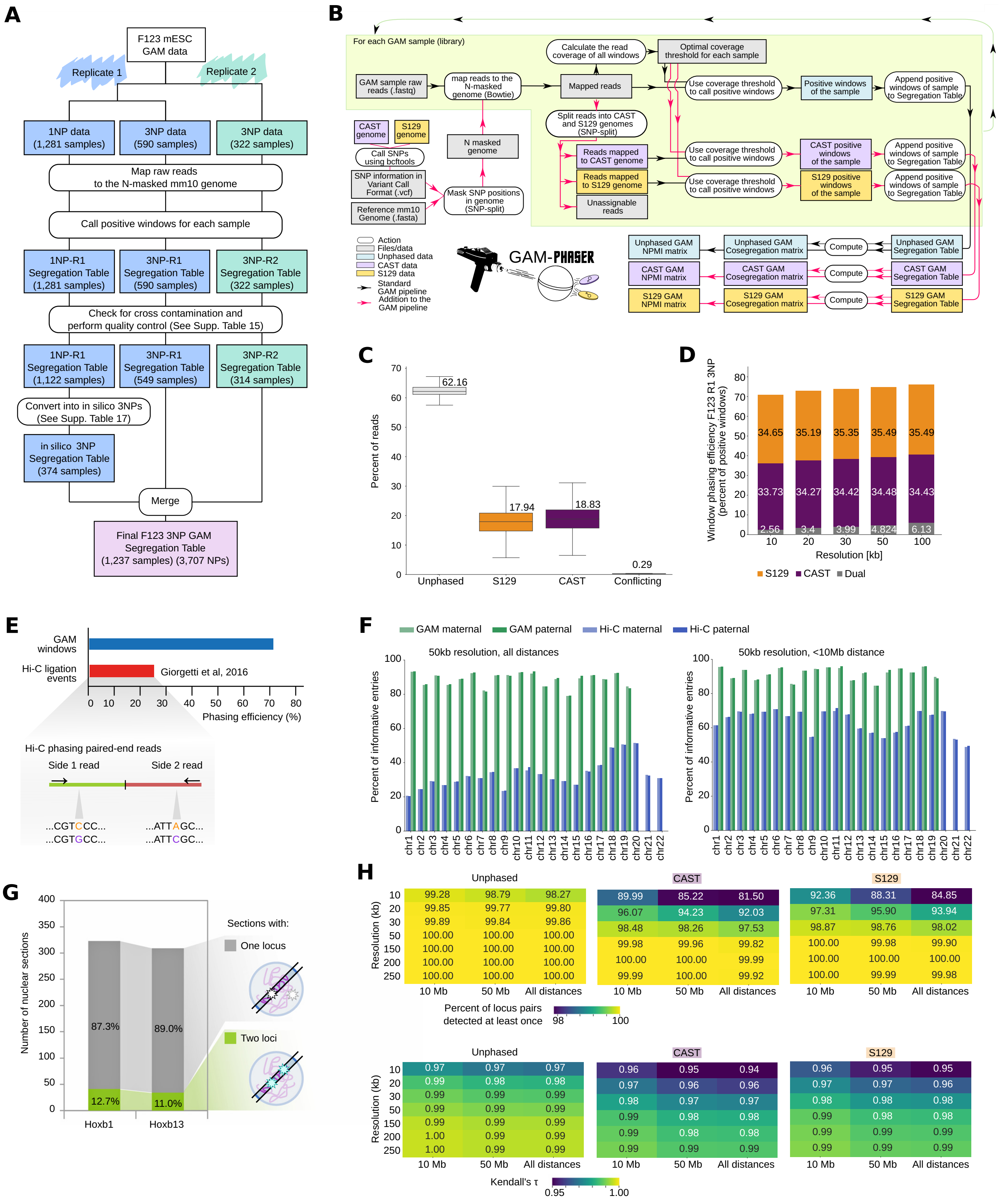

Resolution (kb)	Unphased 10 Mb	Unphased 50 Mb	Unphased All distances	CAST 10 Mb	CAST 50 Mb	CAST All distances	S129 10 Mb	S129 50 Mb	S129 All distances
10	99.28	98.79	98.27	89.99	85.22	81.50	92.36	88.31	84.85
20	99.85	99.77	99.80	96.07	94.23	92.03	97.31	95.90	93.94
30	99.89	99.84	99.86	98.48	98.26	97.53	98.87	98.76	98.02
50	100.00	100.00	100.00	99.98	99.96	99.82	100.00	99.98	99.90
150	100.00	100.00	100.00	100.00	100.00	99.99	100.00	100.00	100.00
200	100.00	100.00	100.00	99.99	100.00	99.92	100.00	99.99	99.98
250	100.00	100.00	100.00						

Resolution (kb)	Unphased 10 Mb	Unphased 50 Mb	Unphased All distances	CAST 10 Mb	CAST 50 Mb	CAST All distances	S129 10 Mb	S129 50 Mb	S129 All distances
10	0.97	0.97	0.97	0.96	0.95	0.94	0.96	0.95	0.95
20	0.99	0.98	0.98	0.97	0.96	0.96	0.97	0.97	0.96
30	0.99	0.99	0.99	0.98	0.97	0.97	0.98	0.98	0.98
50	0.99	0.99	0.99	0.99	0.98	0.98	0.99	0.98	0.98
150	0.99	0.99	0.99	0.99	0.98	0.98	0.99	0.99	0.99
200	1.00	0.99	0.99	0.99	0.99	0.99	0.99	0.99	0.99
250	1.00	0.99	0.99						

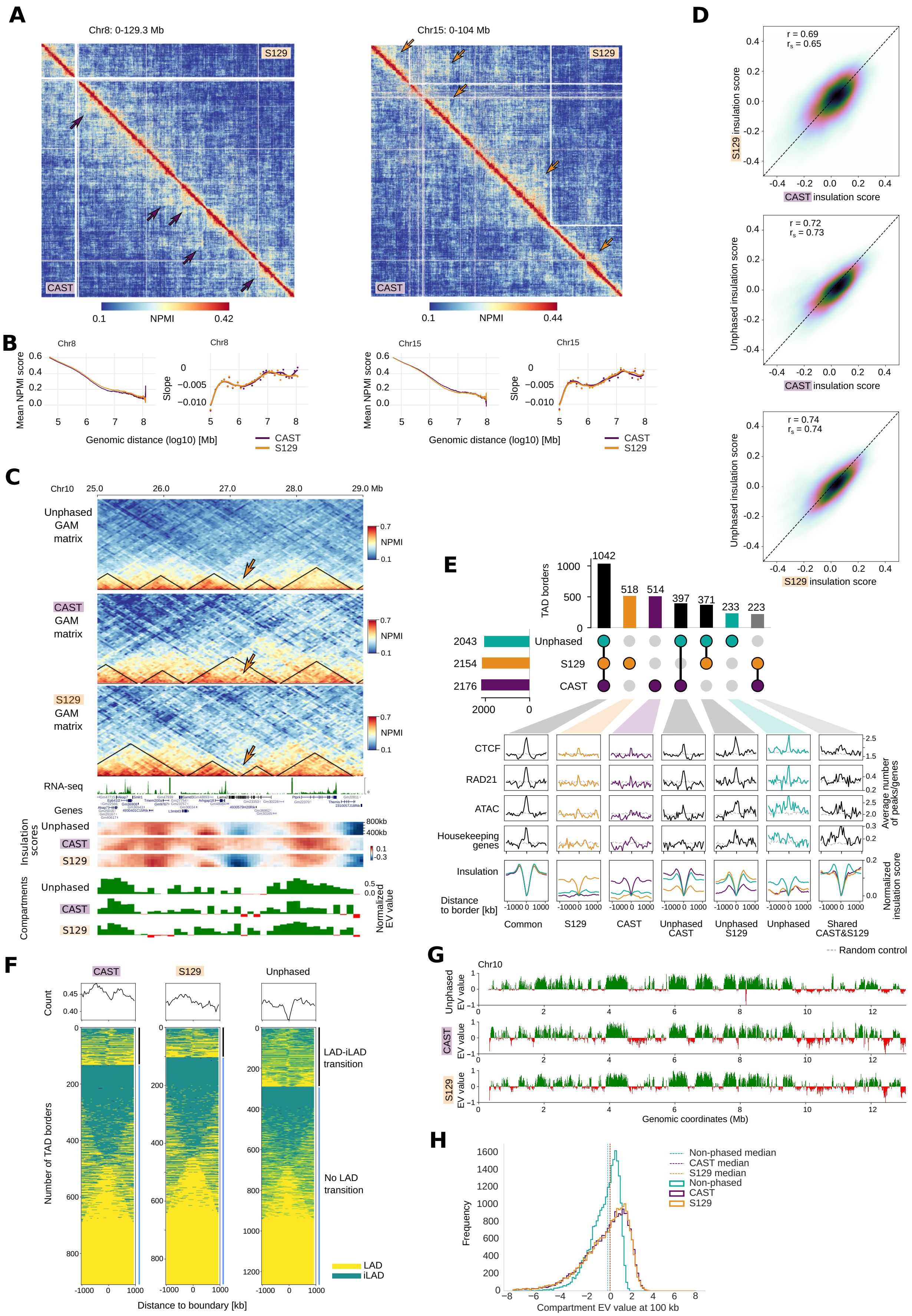
A
Chr8: 0-129.3 Mb
Chr15: 0-104 Mb
S129
CAST
NPMI
B
Mean NPMI score
Slope
Genomic distance (log10) [Mb]
CAST
S129
C
Unphased GAM matrix
CAST GAM matrix
S129 GAM matrix
RNA-seq
Genes
Insulation scores
Compartments
Normalized EV value
D
S129 insulation score
CAST insulation score
Unphased insulation score
r = 0.69
r = 0.72
r = 0.74
E
TAD borders
Unphased
S129
CAST
CTCF
RAD21
ATAC
Housekeeping genes
Insulation
Distance to border [kb]
Common
Unphased CAST
Unphased S129
Shared CAST&S129
Average number of peaks/genes
Normalized insulation score
Random control
F
Count
Number of TAD borders
LAD-iLAD transition
No LAD transition
LAD
iLAD
Distance to boundary [kb]
G
Chr10
Unphased EV value
CAST EV value
S129 EV value
Genomic coordinates (Mb)
H
Frequency
Non-phased median
CAST median
S129 median
Non-phased
Compartment EV value at 100 kb

 © The Author(s)

Figure EV2. Comparison of insulation, TADs and compartments between S129 and CAST haplotypes.

(**A**) GAM matrices of chromosomes 8 and 15 showing both alleles at 50 kb resolution. Colored arrows show structural differences between alleles. (**B**) Distance decay curves and momentum curves for contact intensities across all distances in CAST and S129 chromosomes 8 and 15. (**C**) 4 Mb region in chromosome 10 showing an allele-specific TAD border in the S129 allele. Below, RNA-seq track, insulation scores and compartment tracks for all maps. (**D**) Pearson correlation coefficient (r) between combinations of CAST, S129 and unphased insulation scores at 400 kb. (**E**) Upset plot of TAD border combinations between CAST, S129 and the unphased maps. Below, aggregate plots for CTCF, Rad21 and ATAC-seq peaks and housekeeping genes, centered at the TSS (± 1 kb). Normalized Insulation score is also shown for each group. (**F**) Overlap of LADs and iLADs with ±1,000 kb around CAST, S129 and common TAD borders, computed from 100 kb resolution GAM matrices to match LAD annotations. Each heatmap is clustered depending on whether the border overlaps with a LAD/iLAD transition or not. (**G**) Compartment tracks for CAST, S129 and the unphased maps for chromosome 10. (**H**) Compartment eigenvector values distribution for CAST, S129 and the unphased datasets. Discontinuous lines show the median for each dataset.

© The Author(s)

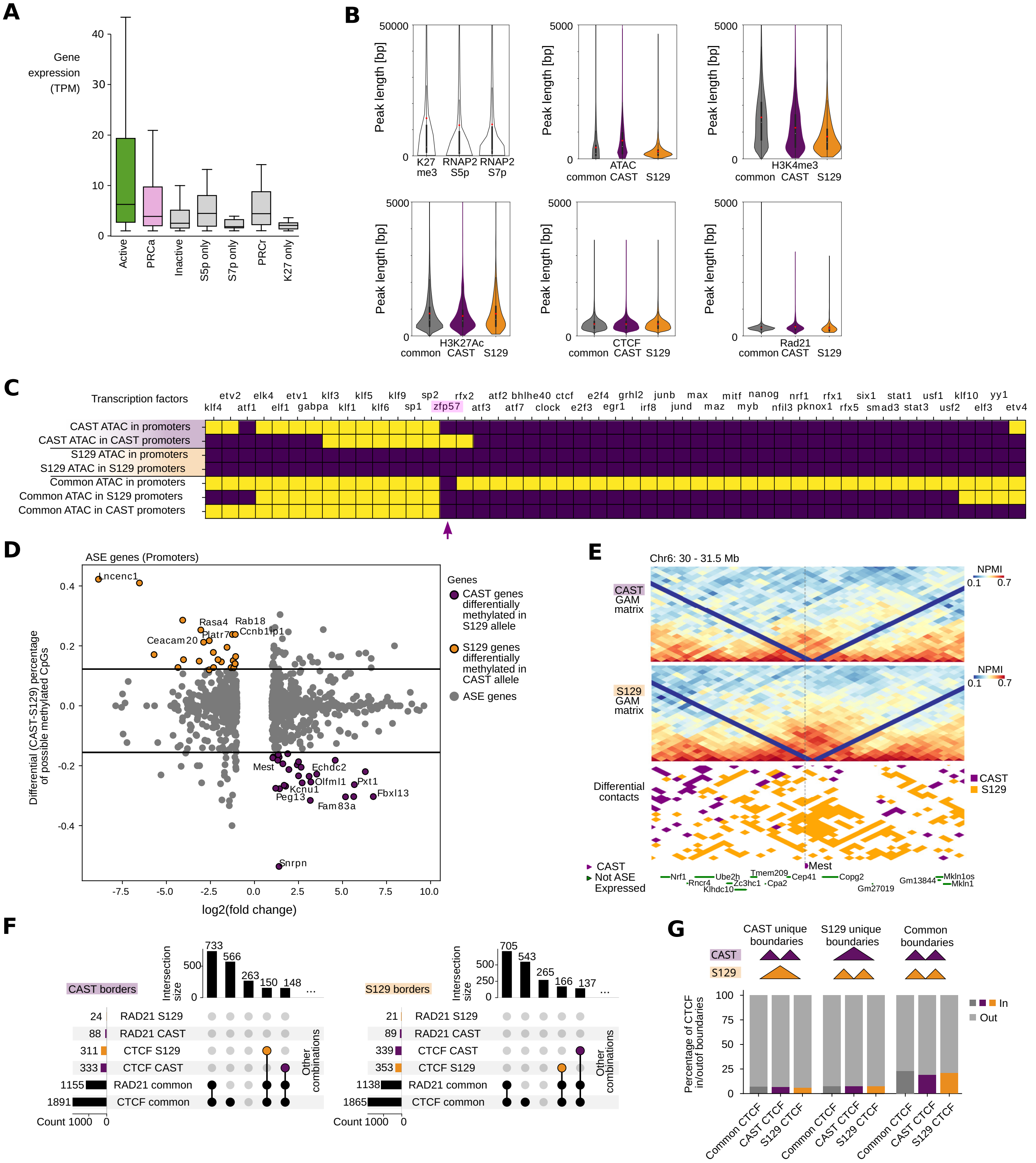

A
Gene expression (TPM)
Active
PRCa
Inactive
S5p only
S7p only
PRCr
K27 only
B
Peak length [bp]
K27 me3
RNAP2 S5p
RNAP2 S7p
ATAC
H3K4me3
H3K27Ac
CTCF
Rad21
common CAST S129
C
Transcription factors
CAST ATAC in promoters
CAST ATAC in CAST promoters
S129 ATAC in promoters
S129 ATAC in S129 promoters
Common ATAC in promoters
Common ATAC in S129 promoters
Common ATAC in CAST promoters
D
ASE genes (Promoters)
Differential (CAST-S129) percentage of possible methylated CpGs
log2(fold change)
Genes
CAST genes differentially methylated in S129 allele
S129 genes differentially methylated in CAST allele
ASE genes
Lncenc1
Rasa4
Rab18
Ccnb1ip1
Platr7
Ceacam20
Mest
Echdc2
Olfml1
Pxt1
Kcnu1
Peg13
Fbxl13
Fam83a
Snrpn
E
Chr6: 30 - 31.5 Mb
CAST GAM matrix
S129 GAM matrix
NPMI
Differential contacts
CAST
Not ASE
Expressed
F
CAST borders
S129 borders
Intersection size
RAD21 S129
RAD21 CAST
CTCF S129
CTCF CAST
RAD21 common
CTCF common
Other combinations
Count
G
CAST unique boundaries
S129 unique boundaries
Common boundaries
Percentage of CTCF in/out of boundaries
In
Out
Common CTCF
CAST CTCF
S129 CTCF

 © The Author(s)

Figure EV3. Association of allele-specific epigenetic marks and transcription factors with promoters of ASE genes and 3D genome organization.

(**A**) Gene expression of the different groups in Fig. 2E. (**B**) Distribution of Pol2-S5p, Pol2-S7p and H3K27me3 peaks, and phased and unphased ATAC-seq, H3K4me3, CTCF, H3K27ac and RAD21 peak sizes. Red dots indicate the average size for each dataset. (**C**) Heatmap showing the enriched presence (cutoffs Q value ≤ 0.05 and *P* value of ≤0.001) of different transcription factors that overlap with the peaks of different ATAC-seq groups. ZFP57 is the only transcription factor enriched for an allele-specific group. (**D**) ASE gene promoters regarding their differential percentage of methylated CpGs. Colored are those genes with a significant amount of methylated CpGs in their promoter (top and bottom 5%) in the allele they are not expressed. (**E**) CAST and S129 GAM matrices for the Mest locus (Chr6: 30–31.5 Mb). Below, differential contacts and two tracks showing CAST genes and expressed genes. (**F**) Most borders contain common CTCF and RAD21 or only CTCF and each allele has a similar number of CTCF specific to either of the alleles in its borders. (**G**) Percentage of CTCF peaks that are inside or outside borders.

© The Author(s)

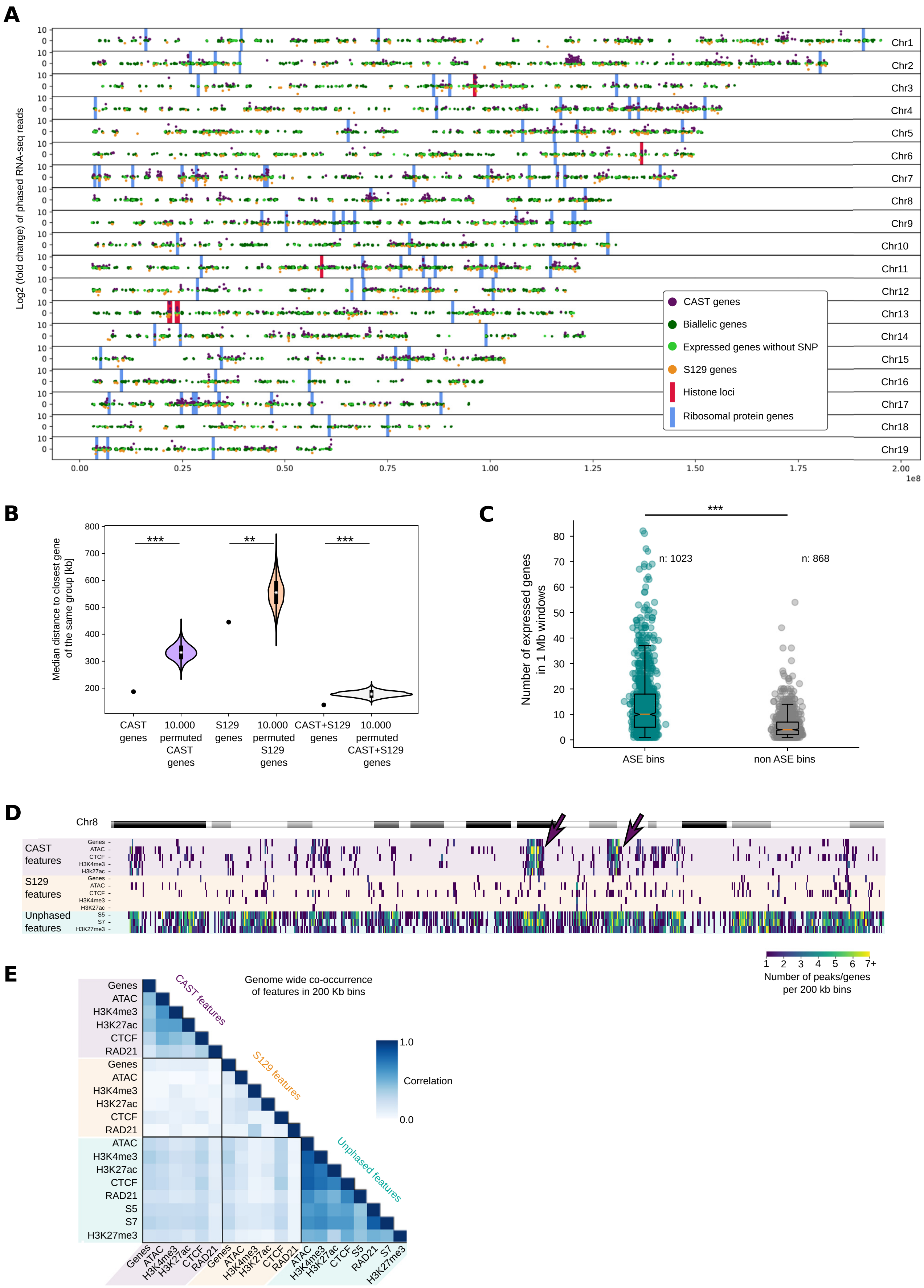
A
Log2 (fold change) of phased RNA-seq reads
Chr1
Chr2
Chr3
Chr4
Chr5
Chr6
Chr7
Chr8
Chr9
Chr10
Chr11
Chr12
Chr13
Chr14
Chr15
Chr16
Chr17
Chr18
Chr19
CAST genes
Biallelic genes
Expressed genes without SNP
S129 genes
Histone loci
Ribosomal protein genes
0.00 0.25 0.50 0.75 1.00 1.25 1.50 1.75 2.00
1e8
B
Median distance to closest gene of the same group [kb]
***
**
***
CAST genes
10.000 permuted CAST genes
S129 genes
10.000 permuted S129 genes
CAST+S129 genes
10.000 permuted CAST+S129 genes
C
Number of expressed genes in 1 Mb windows
***
n: 1023
n: 868
ASE bins
non ASE bins
D
Chr8
CAST features
S129 features
Unphased features
Genes
ATAC
CTCF
H3K4me3
H3K27ac
S5
S7
H3K27me3
1 2 3 4 5 6 7+
Number of peaks/genes per 200 kb bins
E
Genome wide co-occurrence of features in 200 Kb bins
CAST features
S129 features
Unphased features
Genes
ATAC
H3K4me3
H3K27ac
CTCF
RAD21
S5
S7
H3K27me3
1.0
Correlation
0.0

Figure EV4. Co-presence of ASE genes and chromatin features in the linear genome.

(**A**) Genomic position of all expressed genes: CAST genes, S129 genes, biallelic genes and genes without SNPs. Red and Blue bars indicate the position of Histone protein genes and Ribosomal protein genes. (**B**) Each of the 3 dots indicate the average distance of all genes of each type (CAST, S129 and CAST or S129) to the closest gene of that type. The violin plot shows the distribution of these averages if we permute the position of the genes 10,000 times. The permutation is carried out by randomly selecting the same number of CAST, S129 or CAST + S129 genes from all expressed genes. *P* values = 0.0001, 0.0145, 0.0001 for CAST, S129 and CAST + S129. (**C**) 1 Mb windows containing at least 1 ASE gene tend to contain more expressed genes than 1 Mb windows that do not contain ASE genes. T test: *P* value = 2.7×10^{-71}. Number of ASE windows, 1023. Number of non-ASE windows, 868. (**D**) Genomic location of allele-specific features (genes, ATAC-seq, CTCF, H3K4me3 and H3K27ac peaks) and unphased features (Pol2-S5p, Pol2-S7p and H3K27me3 peaks) and their density in bins of 200 kb. Arrows indicate two regions with an enrichment of CAST-specific features. (**E**) Genome-wide Pearson correlation (r) of the co-occurrence of the features in (**D**). CAST features correlate well between each other while S129 features do not.

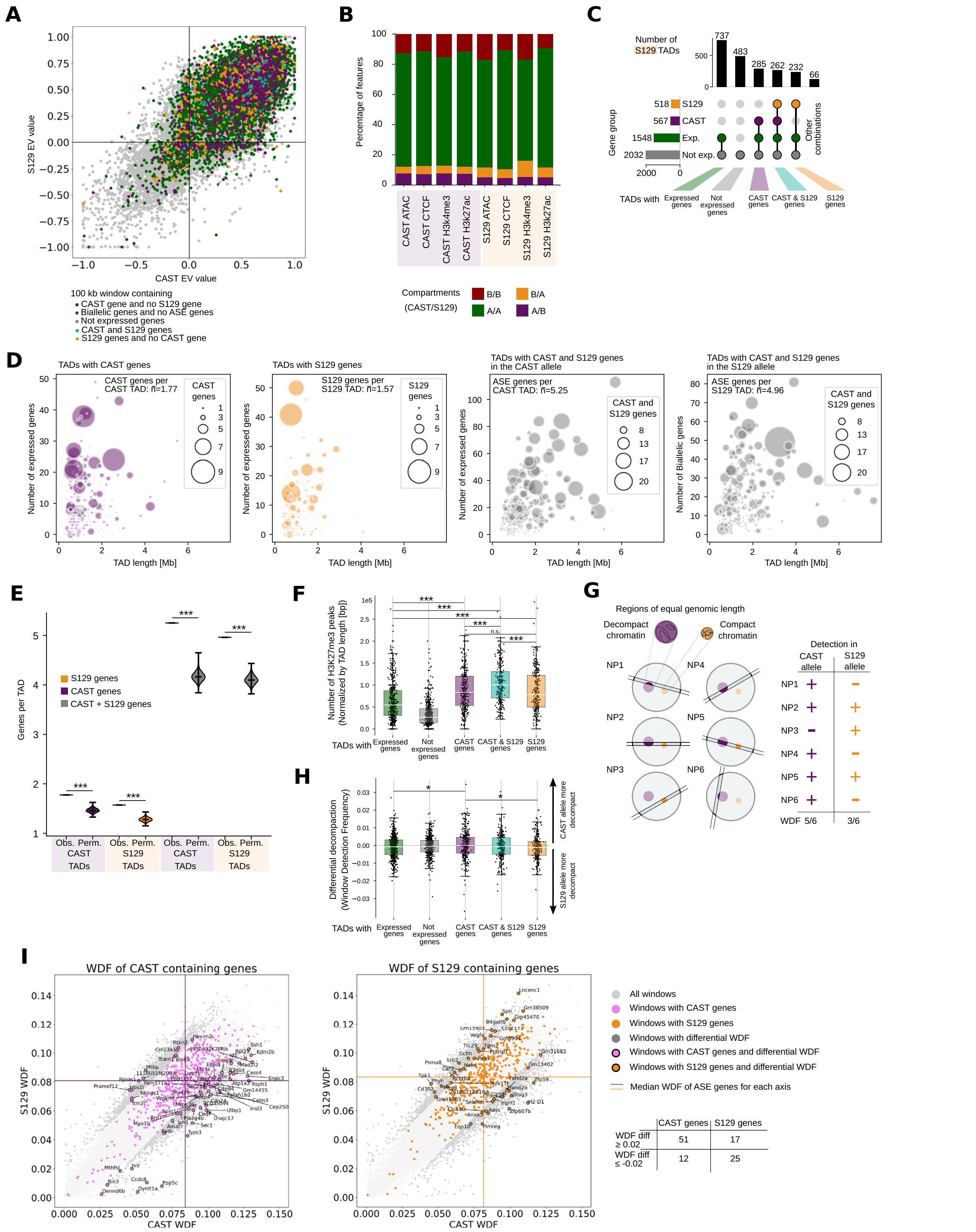

	CAST genes	S129 genes
WDF diff ≥ 0.02	51	17
WDF diff ≤ -0.02	12	25

 © The Author(s)

Figure EV5. Comparative analysis of CAST and S129 alleles in genome compartmentalization and chromatin accessibility.

(**A**) Normalized eigenvector (EV) values for the CAST and S129 allele for each 100 kb bin. Color coded are bins containing only not expressed genes, bins containing biallelic genes but not ASE genes, bins containing at least one CAST gene but not S129, bins containing at least one S129 gene but not CAST genes and bins containing at least one CAST gene and one S129 gene. (**B**) Percentage of ATAC-seq, CTCF, H3K4me3 or H3K27ac peaks in each compartment combination (A and A, B and B, A and B or B and A for CAST and S129 alleles, respectively). CAST-specific features show a tendency to overlap more in A/B (A specific compartment in the CAST allele), S129-specific features tend to overlap more in B/A (A specific compartment in the S129 allele.). (**C**) UpSet plots showing for the S129 allele, groups of TADs containing different sets of types of genes and their number. (**D**) Relation between the TAD length, the number of expressed genes in a TAD, and number of genes specific to that allele (dot size) for TADs in CAST and S129. Purple refers to TADs containing CAST genes, orange to TADs containing S129 genes, and gray to TADs containing CAST and S129 genes (for both CAST allele and S129 allele, respectively). (**E**) Violin plots showing the number of genes per TAD (observed, Obs.) compared to circular permutations of gene positions in the genome (permuted, Perm.). 10,000 permutations were done for each of the 4 examples in (**D**) and are compared to the number of genes per TAD in the original data (called *real*). All P values are ≤ 0.0001. Numbers are 911, 889, 1265 and 1265, respectively. (**F**) Related to (**C**), number of H3K27me3 peaks normalized by TAD length (two-sided t test: $*P \leq 0.05$, $**P \leq 0.01$, $***P \leq 0.001$; P values from top to bottom in S129 TADs: 1.9×10^{-14}, 3.2×10^{-30}, 1.1×10^{-10}, 0.00011, 1.8×10^{-5}, n.s: 0.5092. Number of TADs with: expressed genes, 737; not expressed genes, 483; CAST genes, 285; S129 genes, 232; and with CAST and S129 genes, 262). (**G**) Loci with the same genomic length can have different volumes due to varying compaction. Decompacted loci with larger volumes are captured more frequently in the collection of GAM cryosections than more compacted chromatin. Window Detection Frequency (WDF) is a GAM-intrinsic measure of relative chromatin compaction, defined by the number of locus detection events in the collection of GAM nuclear slices (Beagrie et al, 2017). From phased window segregation tables, the WDF can be calculated separately for CAST and S129, as a measure of relative compaction between all loci in each haplotype. (**H**) Related to (**C**), for each group, the differential (CAST-S129) window detection frequency is represented. Positive values indicate decompaction in the CAST allele, while negative values indicate decompaction in the S129 allele (two-sided t test: $*P \leq 0.05$, $**P \leq 0.01$, $***P \leq 0.001$; P values from top to bottom for S129 TADs: 0.031, 0.025). Number of TADs analyzed are the same as in (**F**). (**I**) Window detection frequency (WDF) values in the CAST and S129 allele for each bin containing genes. Fisher's exact test ($P = 5.3 \times 10^{-5}$) shows the significant tendency of windows with high WDF in the CAST allele containing CAST genes and windows with high WDF in the S129 allele containing S129 genes compared to windows with lower WDF. Numbers for CAST and S129 genes with differential WDF ≥ 0.02 are 51 and 17, respectively. For CAST and S129 genes with differential WDF ≤ -0.02 are 12 and 25, respectively.

© The Author(s)

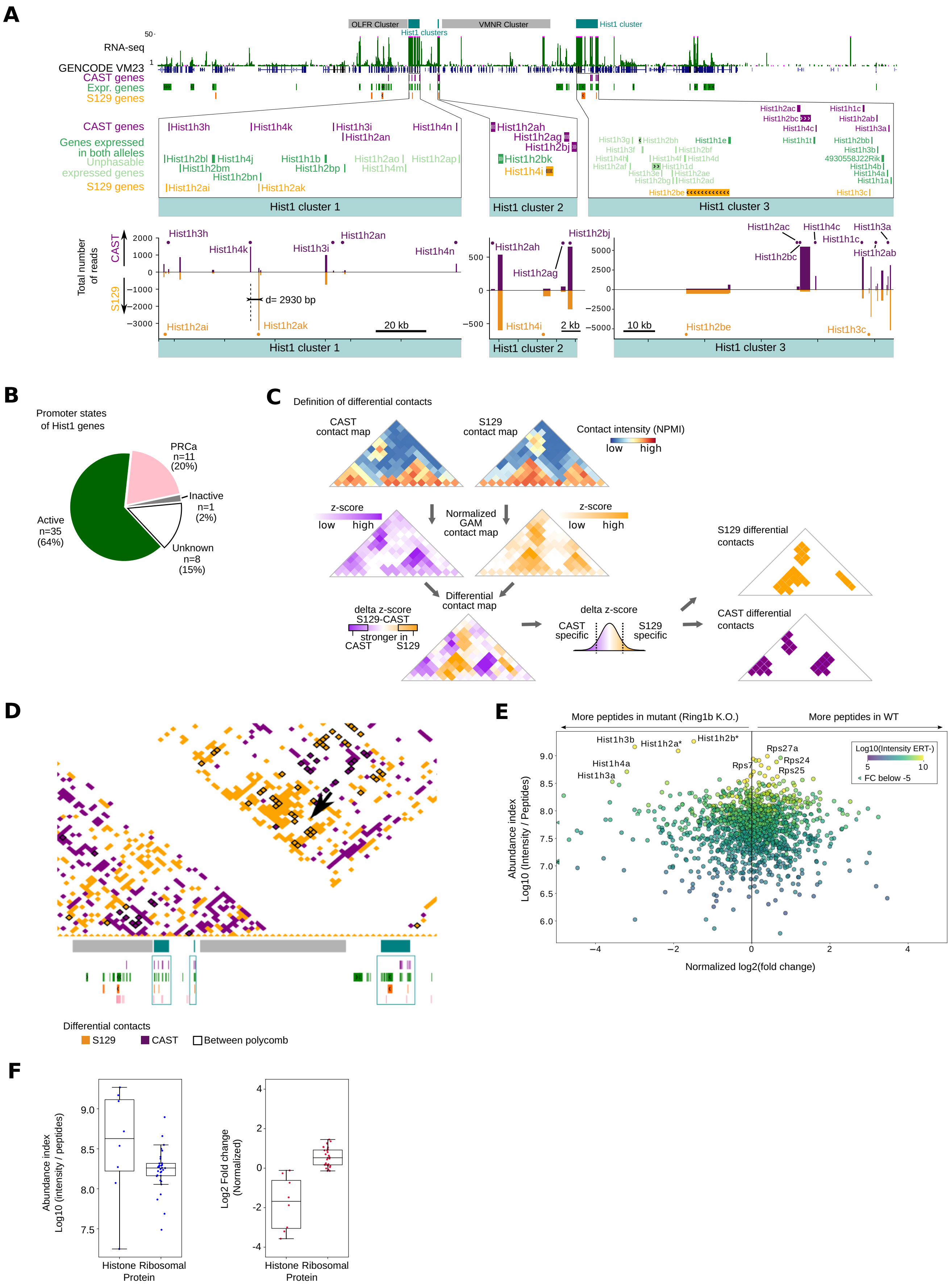
A
OLFR Cluster
Hist1 clusters
VMNR Cluster
Hist1 cluster
RNA-seq
GENCODE VM23
CAST genes
Expr. genes
S129 genes
CAST genes
Genes expressed in both alleles
Unphasable expressed genes
S129 genes
Hist1h3h
Hist1h4k
Hist1h3i
Hist1h2an
Hist1h4n
Hist1h2bl
Hist1h4j
Hist1h2bm
Hist1h2bn
Hist1h1b
Hist1h2bp
Hist1h2ao
Hist1h4m
Hist1h2ap
Hist1h2ai
Hist1h2ak
Hist1h2ah
Hist1h2ag
Hist1h2bj
Hist1h2bk
Hist1h4i
Hist1h2ac
Hist1h2bc
Hist1h4c
Hist1h1c
Hist1h2ab
Hist1h3a
Hist1h3g
Hist1h2bh
Hist1h1e
Hist1h1t
Hist1h2bb
Hist1h3f
Hist1h2bf
Hist1h3b
Hist1h4h
Hist1h4f
Hist1h4d
4930558J22Rik
Hist1h2af
Hist1h1d
Hist1h4b
Hist1h3e
Hist1h2ae
Hist1h4a
Hist1h2bg
Hist1h2ad
Hist1h1a
Hist1h2be
Hist1h3c
Hist1 cluster 1
Hist1 cluster 2
Hist1 cluster 3
Total number of reads
CAST
S129
d= 2930 bp
20 kb
2 kb
10 kb
B
Promoter states of Hist1 genes
PRCa n=11 (20%)
Inactive n=1 (2%)
Unknown n=8 (15%)
Active n=35 (64%)
C
Definition of differential contacts
CAST contact map
S129 contact map
Contact intensity (NPMI)
low high
z-score
low high
Normalized GAM contact map
S129 differential contacts
Differential contact map
delta z-score S129-CAST
stronger in CAST S129
delta z-score
CAST specific
S129 specific
CAST differential contacts
D
Differential contacts
S129
CAST
Between polycomb
E
More peptides in mutant (Ring1b K.O.)
More peptides in WT
Hist1h3b
Hist1h2a*
Hist1h2b*
Hist1h4a
Hist1h3a
Rps27a
Rps24
Rps7
Rps25
Log10(Intensity ERT-)
FC below -5
Abundance index Log10 (Intensity / Peptides)
Normalized log2(fold change)
F
Abundance index Log10 (intensity / peptides)
Histone Ribosomal Protein
Log2 Fold change (Normalized)
Histone Ribosomal Protein

 © The Author(s)

Figure EV6. Differential gene regulation at the Hist1 gene cluster.

(**A**) Hist1 locus region from Fig. 4A with greater detail. Histone genes are depicted. Number of reads phased for the CAST or S129 allele are shown for all genes in the Hist1 clusters. (**B**) Proportion of Hist1 genes according to promoter state. (**C**) Schematic showing the pipeline used to compare CAST and S129 contact intensities and to extract CAST-specific and S129-specific contacts. (**D**) Allele-specific contacts at the Hist1 locus as shown in Fig. 4A. Black squares show the contacts where H3K27me3 peaks are present in both windows of the contact. (**E**) SILAC experiments were performed in the ESC-ERT2 cells in the presence and absence of tamoxifen to induce knockout of *Ring1b*, in three biological replicates. *Ring1b* knockout results in upregulation of histone proteins. Abundance was estimated by the ratio of intensity and number of peptides. Normalized log2 fold change was calculated applying the z-score normalization to the log2 of heavy/light (H/L) ratio of the untreated experiment divided by the H/L ratio of the conditional knockout. (**F**) Boxplots showing the abundance index and the log2 fold change for detected histone proteins and ribosomal proteins. Numbers of data points are 8, 32, 8, 32, respectively from left to right.

© The Author(s)

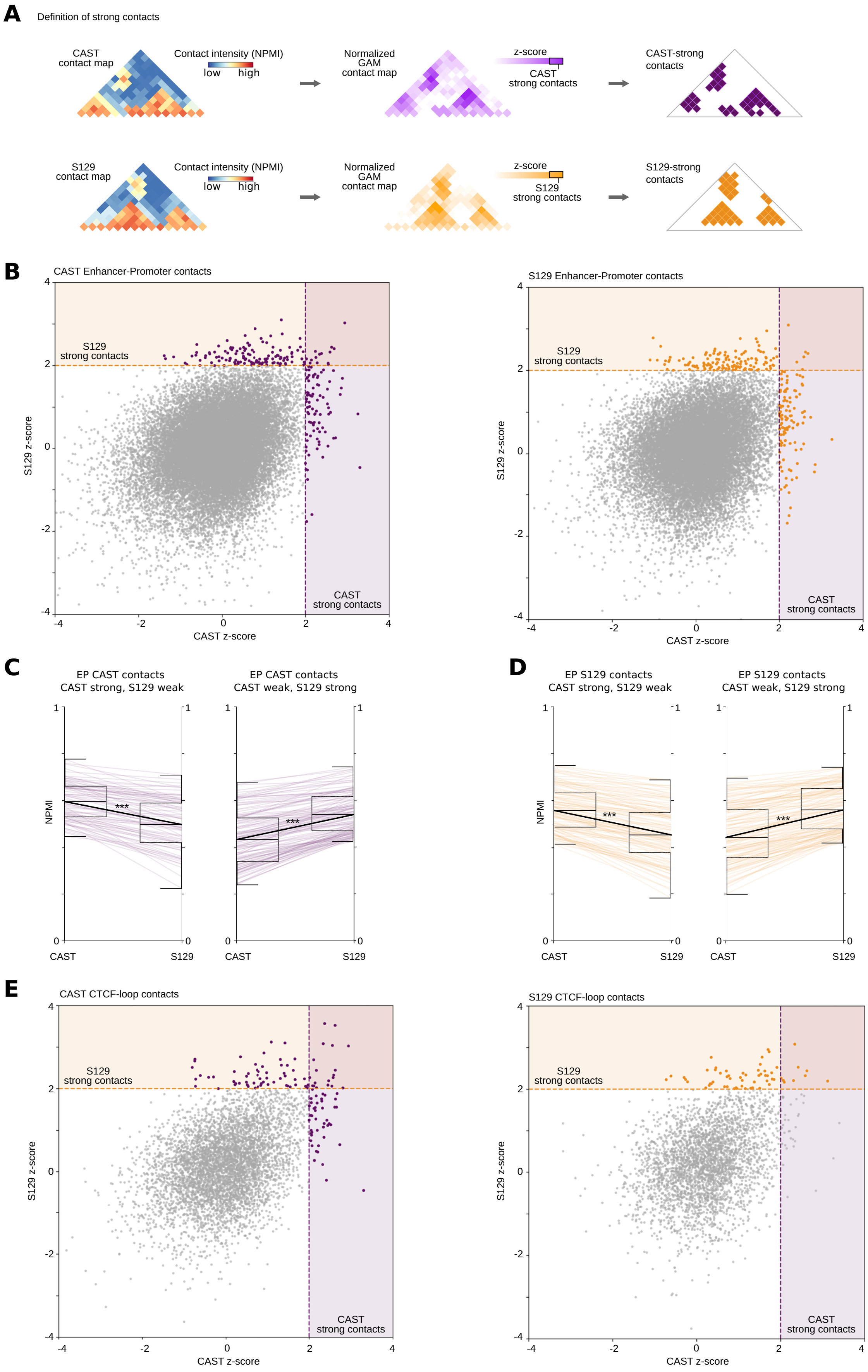
A
Definition of strong contacts
CAST
contact map
Contact intensity (NPMI)
low high
Normalized
GAM
contact map
z-score
CAST
strong contacts
CAST-strong
contacts
S129
contact map
Contact intensity (NPMI)
low high
Normalized
GAM
contact map
z-score
S129
strong contacts
S129-strong
contacts
B
CAST Enhancer-Promoter contacts
S129 Enhancer-Promoter contacts
S129
strong contacts
CAST
strong contacts
S129 z-score
CAST z-score
C
EP CAST contacts
CAST strong, S129 weak
EP CAST contacts
CAST weak, S129 strong
NPMI
***
CAST
S129
D
EP S129 contacts
CAST strong, S129 weak
EP S129 contacts
CAST weak, S129 strong
E
CAST CTCF-loop contacts
S129 CTCF-loop contacts

 © The Author(s)

Figure EV7. Strong allelic contacts bridge promoters, enhancers, and CTCF sites.

(**A**) Schematic showing the strategy to identify strong allelic contacts. (**B**) All possible contacts involving conditions for CAST-specific enhancer–promoter contacts and S129-specific enhancer–promoter contacts. Lines mark cutoffs for strong and allele-specific contacts in each haplotype. (**C**) Differences in contact intensities observed in the CAST and S129 haplotypes for allele-specific enhancer–promoter (E–P) elements associated with CAST contacts that were found to be strong in CAST but weak in S129 (on the left), or strong in S129 but weak in CAST (on the right). Two sample *t* test: *P* values are 4.17e-10 and 8.78e-17, respectively. Numbers are 84 and 130 respectively. (**E**) All possible contacts involving conditions for CAST-specific CTCF loops and S129-specific CTCF loops. Lines mark cutoffs for strong and allele-specific contacts in each haplotype. (**D**) Differences in contact intensities observed in the CAST and S129 haplotypes for allele-specific enhancer–promoter (E–P) elements associated with S129 contacts that were found to be strong in CAST but weak in S129 (on the left), or strong in S129 but weak in CAST (on the right). Two sample t test: p values are 3.92e-12 and 4.42e-14, respectively. Numbers are 112 and 129, respectively.

© The Author(s)

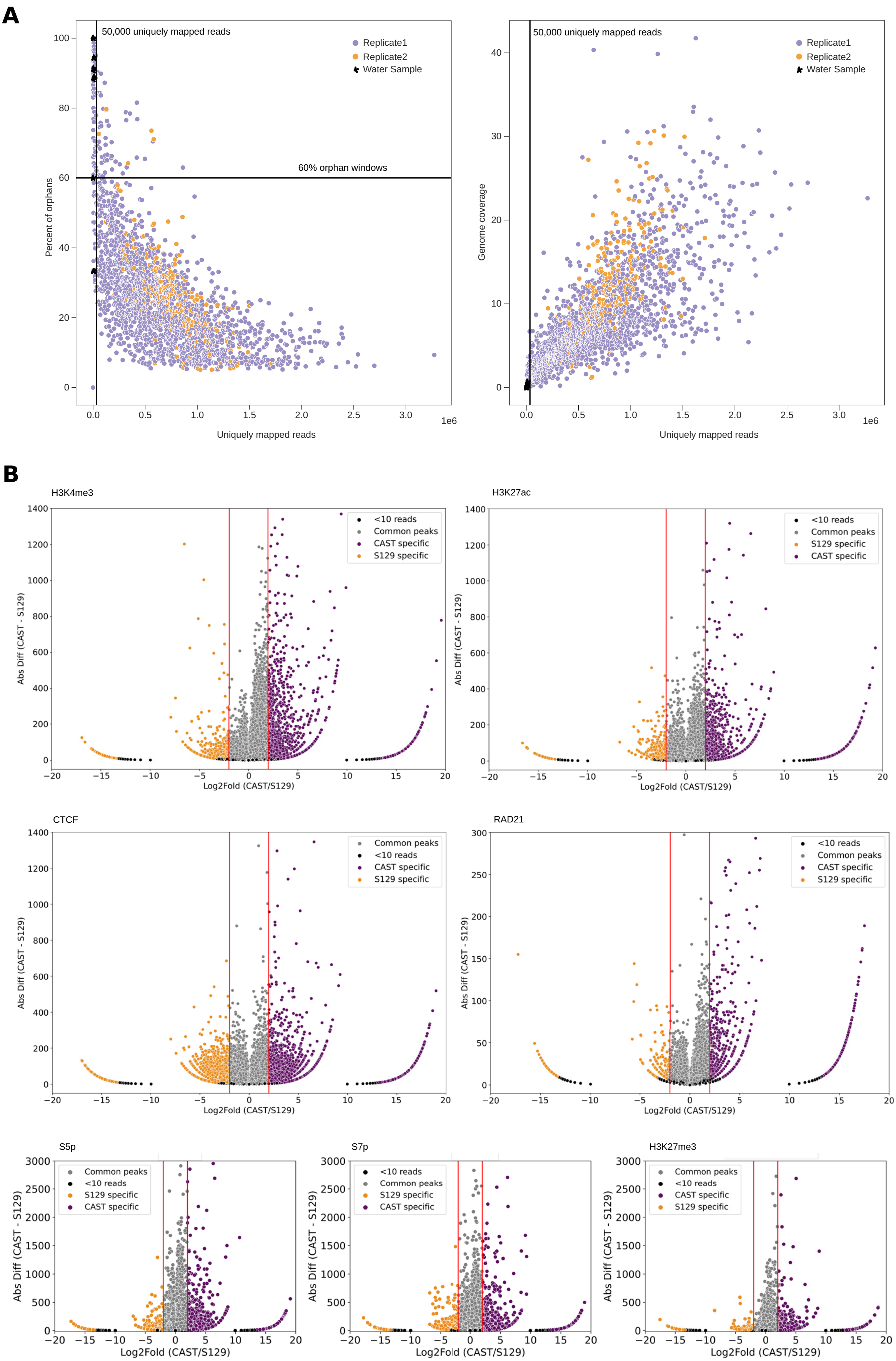
A
50,000 uniquely mapped reads
Replicate1
Replicate2
Water Sample
60% orphan windows
Percent of orphans
Uniquely mapped reads
1e6
50,000 uniquely mapped reads
Replicate1
Replicate2
Water Sample
Genome coverage
Uniquely mapped reads
1e6
B
H3K4me3
<10 reads
Common peaks
CAST specific
S129 specific
Abs Diff (CAST - S129)
Log2Fold (CAST/S129)
H3K27ac
<10 reads
Common peaks
S129 specific
CAST specific
Abs Diff (CAST - S129)
Log2Fold (CAST/S129)
CTCF
Common peaks
<10 reads
CAST specific
S129 specific
Abs Diff (CAST - S129)
Log2Fold (CAST/S129)
RAD21
<10 reads
Common peaks
CAST specific
S129 specific
Abs Diff (CAST - S129)
Log2Fold (CAST/S129)
S5p
Common peaks
<10 reads
S129 specific
CAST specific
Abs Diff (CAST - S129)
Log2Fold (CAST/S129)
S7p
<10 reads
Common peaks
S129 specific
CAST specific
Abs Diff (CAST - S129)
Log2Fold (CAST/S129)
H3K27me3
Common peaks
<10 reads
CAST specific
S129 specific
Abs Diff (CAST - S129)
Log2Fold (CAST/S129)

© The Author(s)

Figure EV8. Assessment of read-count thresholds in phasing.

(**A**) Distribution of percentage of orphan windows, uniquely mapped reads and genome coverage in each GAM sample. Replicate 1, replicate 2 and water (0NP) samples are shown. Thresholds used to remove potentially low from high quality GAM samples are shown in vertical and horizontal black lines. (**B**) Distribution of phased H3K4me3, H3K27ac, CTCF, RAD21, Pol2-S5p, Pol2-S7p, and H3K27me3 peaks, showing their absolute difference in phased reads (CAST-S129) and their log2 fold change.
